# Supplementary material for: The evolution of organic material on Asteroid 162173 Ryugu and its delivery to Earth
Source: Nat Commun. 2024 Jul 22;15:6165. doi: 10.1038/s41467-024-50004-w (PMC11263614; doi:10.1038/s41467-024-50004-w)
Supplement: Supplementary file 1 — Supplementary Information [file 41467_2024_50004_MOESM1_ESM.pdf]

The evolution of organic material on Asteroid 162173 Ryugu and its delivery to Earth: Supplementary Material

H. G. Changela,[1,2] Y. Kebukawa,[3,4] L. Petera,[1,5] M. Ferus,[1] E. Chatzitheodoridis,[6,7] L. Nejdl,[8] R. Nebel,[1] V. Protiva,[1] P. Krepelka,[9] J. Moravcova,[9] R. Holbova,[9] Z. Hlavenkova,[9] T. Samoril,[10,11] J. C. Bridges,[12] S. Yamashita,[13] Y. Takahashi,[14] T. Yada,[15] A. Nakato,[15] K. Sobotkova,[11] H. Tesarova,[11] and D. Zapotok[16]

1. J. Heyrovsky Institute of Physical Chemistry, Czech Academy of Sciences, Prague, Czechia. 2. Department of Earth & Planetary Sciences, University of New Mexico, Albuquerque, New Mexico, USA. 3. Department of Chemistry and Life Science, Yokohama National University, Yokohama, Japan. 4. Department of Earth and Planetary Sciences, Tokyo Institute of Technology, Tokyo, Japan. 5. Department of Inorganic Chemistry, Faculty of Science, Charles University, Prague, Czechia. 6. National Technical University of Athens, Greece. 7. ESTEC, European Space Agency, Noordwijk, The Netherlands. 8. Department of Chemistry and Biochemistry, Mendel University, Brno, Czechia. 9. Central European Institute of Technology Masaryk University, Brno, Czechia. 10. Central European Institute of Technology, Brno University of Technology, Czechia. 11. TESCAN GROUP a.s., Brno, Czechia. 12. Space Park Leicester, School of Physics & Astronomy, University of Leicester, Leicester, UK. 13. Institute of Materials Structure Science, High-Energy Accelerator Research Organization, Ibaraki, Japan. 14. Department of Earth and Planetary Science, The University of Tokyo, Tokyo, Japan. 15. Astromaterials Science Research Group, Institute of Space and Astronautical Science, Japan Aerospace Exploration Agency, Kanagawa, Japan. 16. TESCAN USA Inc, Pennsylvania, USA. Email: changela@unm.edu

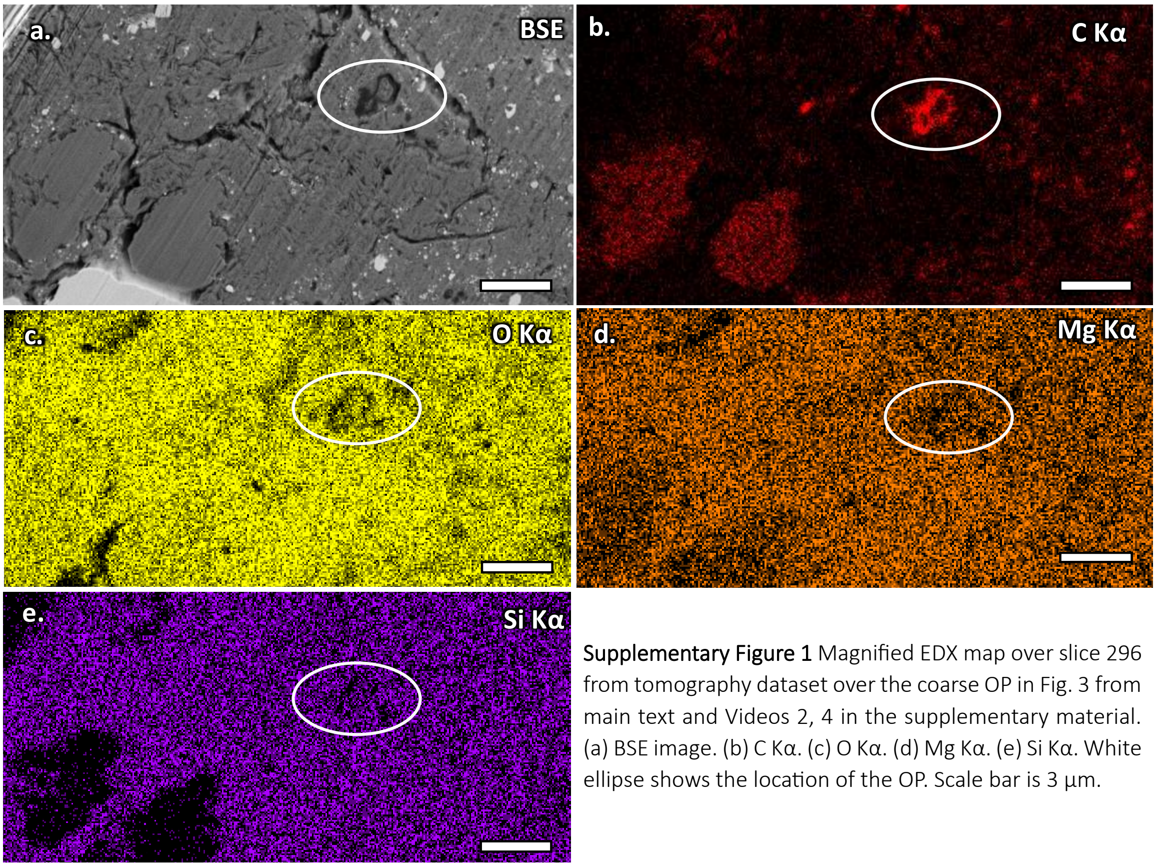

Supplementary Figure 1 Magnified EDX map over slice 296 from tomography dataset over the coarse OP in Fig. 3 from main text and Videos 2, 4 in the supplementary material. (a) BSE image. (b) C Kα. (c) O Kα. (d) Mg Kα. (e) Si Kα. White ellipse shows the location of the OP. Scale bar is 3 µm.

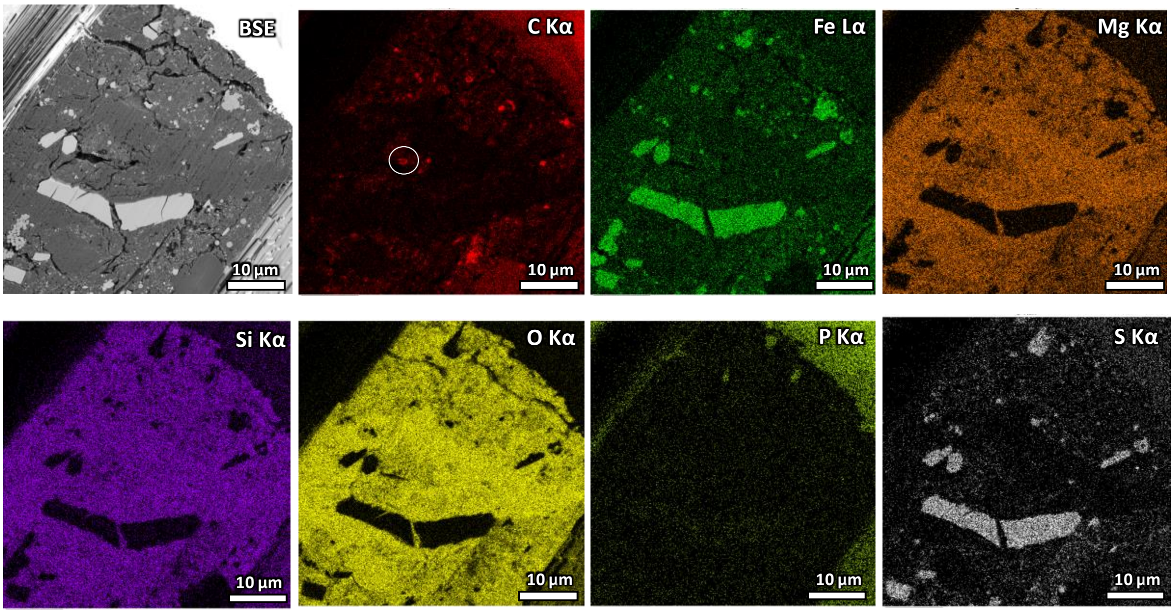

Supplementary Figure 2 EDX maps from slice 420 (Fig. 4 in main text). Red is C Kα. White circle is the OP in Fig. 4 from the manuscript and Supplementary Movies 3 and 4.

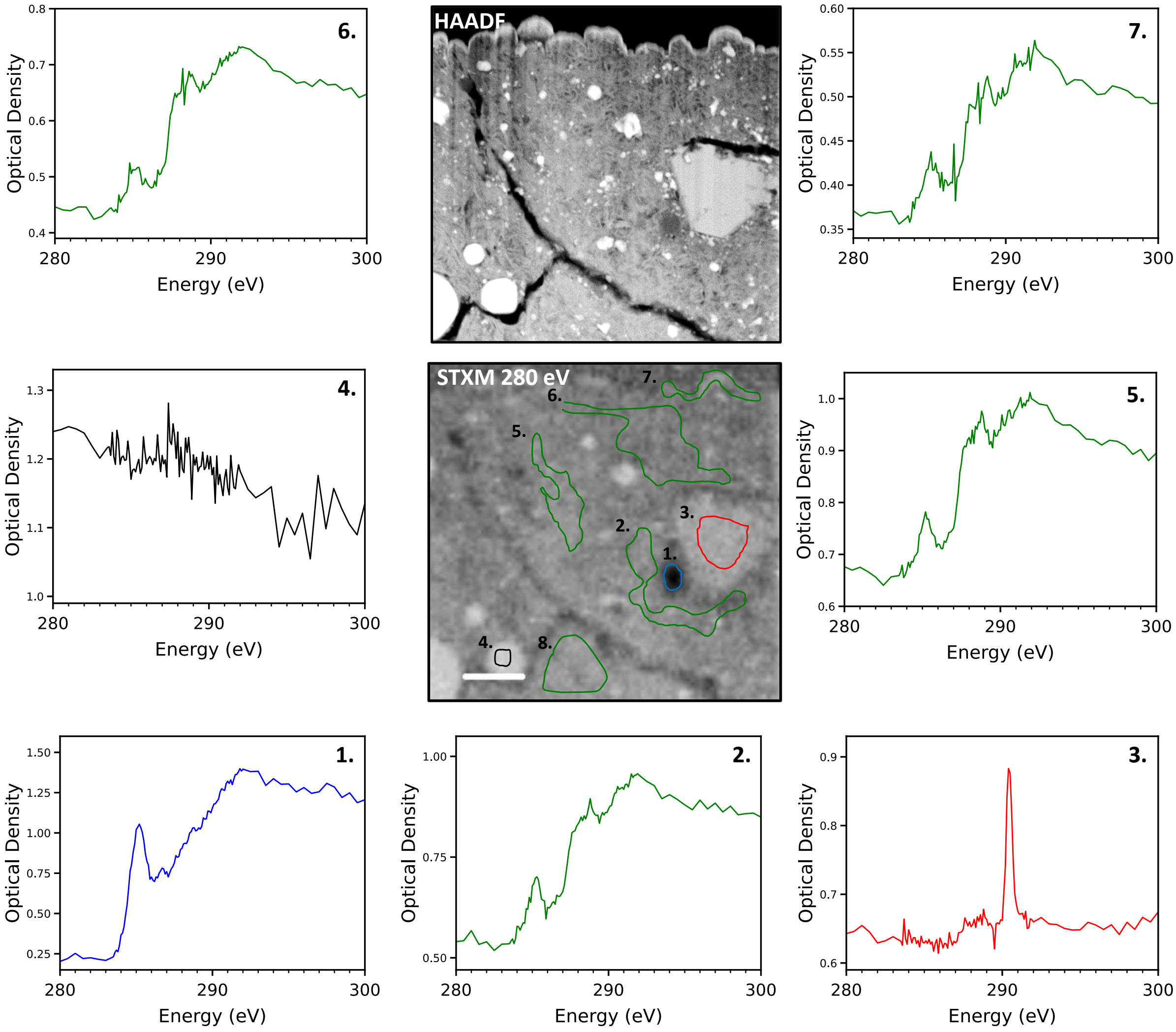

Supplementary Figure 3 XANES of ROIs from A0083 Xe^+ lamella (Figure 6 in main text). Top image is HAADF image of the region of the lamella where the XANES stack was made. Below is the 280 eV OD image. ROI 1 is the OP. 2 is the surrounding diffuse OM. 3 is the carbonate grain adjacent to the OP. 4 is the Fe-rich grain (probably magnetite framboid). 5-7 are diffuse OM ROIs. Note the ROI no. 7 at the bottom of the lamella, where a tentative peak at ~290.3 eV was formed probably due to radiolysis by the lamella sample preparation. Scale bar is 2 µm.

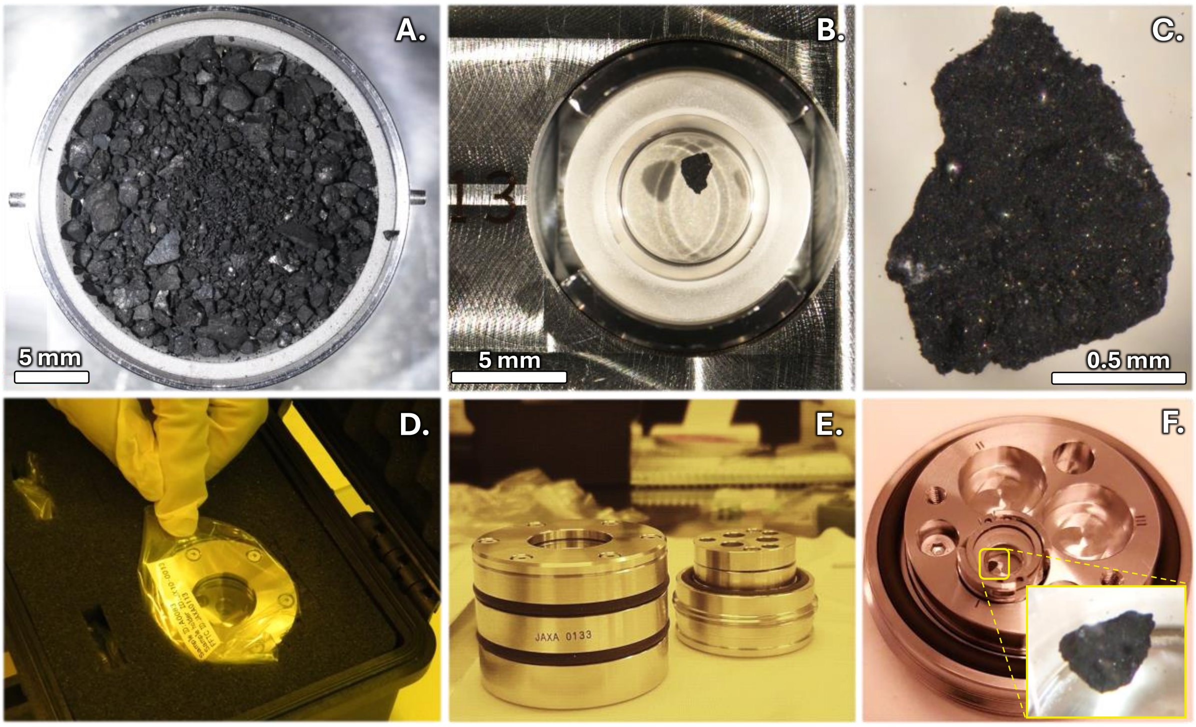

Supplementary Figure 4. Grain A0083 (Radegast). (A) Photograph of Hayabusa-2 collection chamber from JAXA. (B) Image of grain A0083 from the JAXA Hayabusa-2 sample database system https://darts.isas.jaxa.jp/curation/hayabusa2/. (C) Higher magnification of A0083. (D) A0083 delivered in Facility-to-Facility Transfer Container (FFTC). (E) Opened FFTC. (F) Inside of the FFTC. The sample was placed on a sapphire dish with a quartz window screwed over the top of it.

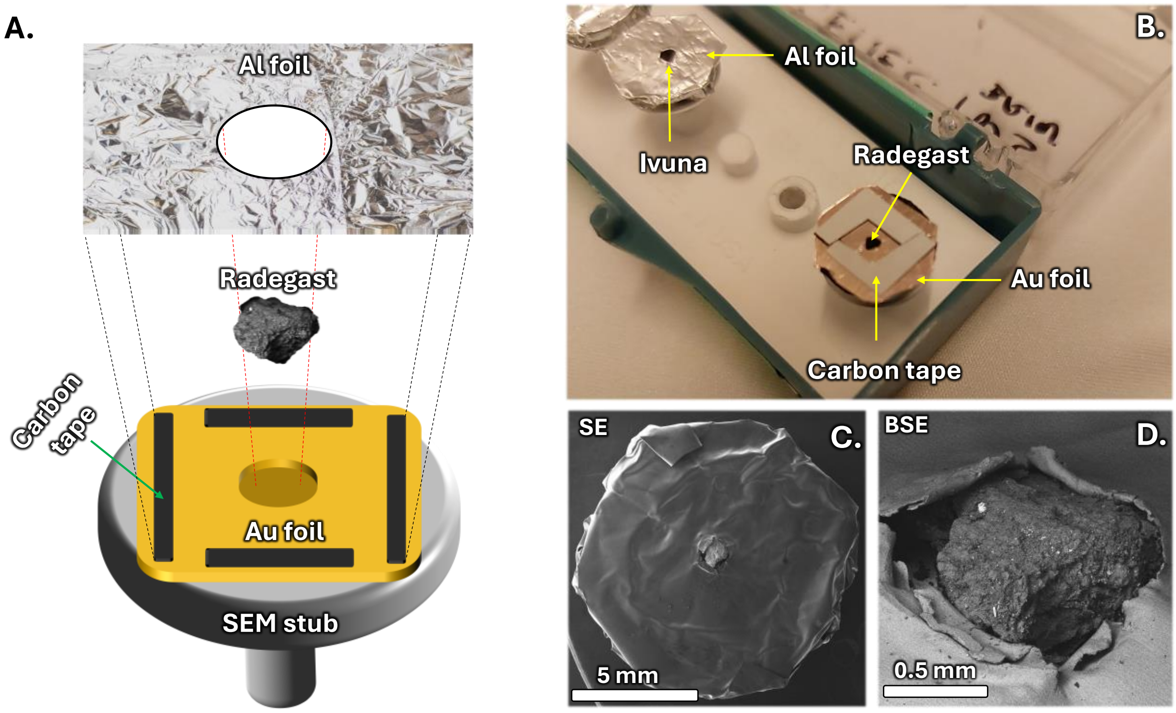

Supplementary Figure 5. Preparation of A0083 (Radegast). (A) Schematic of apparatus used for holding the grain as described in the methods section. (B) Image of the grain prior to placing of the Al foil over the top of it. (C) Low kV SE image of the grain after placement of the foil over the top of it. (D) Higher magnification image of the grain protruding from the hole in the foil.

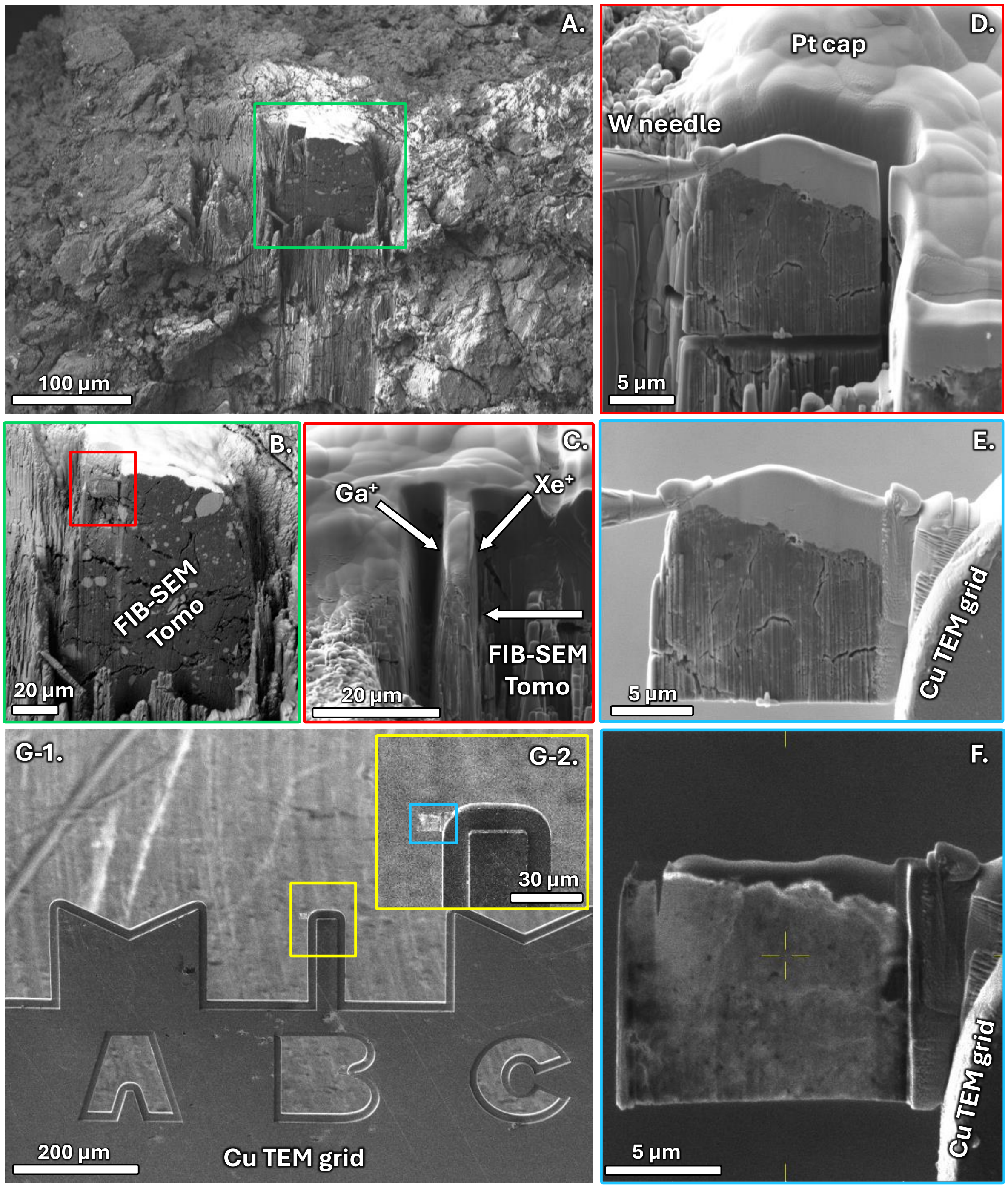

Supplementary Figure 6 Lamella extraction at the end of the tomography region with the LYRA3 (Ga^+ FIB source). (A) BSE image of the edge of A0083 where the tomography volume and lamella extractions were made. (B) Region where the lamella extractions were made. (C) Rotated BSE side view of the FIB lamella extractions with the Xe^+ plasma PFIB (AMBER X) and Ga^+ FIB (LYRA3 already extracted). (D) Extraction of the lamella with the LYRA3. (E) Attachment of the lamella to the TEM Cu grid. (F) 5kV snap shot of the final lamella after polishing at low currents. (G) Low mag image of the lamella on the Cu TEM grid.
