# Supplementary material for: The evolution of organic material on Asteroid 162173 Ryugu and its delivery to Earth
Source: Nat Commun. 2024 Jul 22;15:6165. doi: 10.1038/s41467-024-50004-w (PMC11263614; doi:10.1038/s41467-024-50004-w)
Supplement: Supplementary file 3 — Description of Additional Supplementary Files [file 41467_2024_50004_MOESM3_ESM.pdf]

Description of Additional Supplementary Files

File Name: Supplementary Movie 1
Description: FIB-SEM tomography over the entire volume. Each BSE image is 2056x2056 pixels with resolution 25 nm/pixel. The slice depth is 25 nm. 325 slices are in this tomography set (~8.125 µm depth), with each image size 51.4 x 51.4 µm.

File Name: Supplementary Movie 2
Description: FIB-SEM tomography magnified from dataset in Video 1 over coarsest organic particle (OP) in BSE. Slice and view is played forwards and backwards.

File Name: Supplementary Movie 3
Description: FIB-SEM tomography magnified from dataset in Video 1 in BSE, over OP surrounding the silicate matrix material. Slice and view is played forwards and backwards.

File Name: Supplementary Movie 4
Description: 3D Render of both OPs in in videos 2 and 3. Purple is the organic segment (dark grey from BSE tomography). Green is the lighter grey silicate matrix segment.
